# Management of pediatric testicular microlithiasis

**DOI:** 10.3389/fped.2025.1588261

**Published:** 2025-08-20

**Authors:** Hunter A. Flores, Walker C. Bailey, Kelly T. Harris

**Affiliations:** ^1^ Children’s Hospital Colorado, Aurora, CO, United States; ^2^ School of Medicine, University of Colorado Anschutz Medical Campus, Aurora, CO, United States

**Keywords:** testicular microlithiasis, pediatric urology, pediatric screening, testicular abnormalities, urological abnormalities

## Abstract

Testicular microlithiasis (TM) is a primarily asymptomatic condition characterized by the accumulation of microscopic calcium deposits within the seminiferous tubules. While typically identified incidentally on ultrasonography, TM has generated clinical interest due to its potential links to infertility and testicular malignancy. TM is also associated with benign conditions like cryptorchidism, varicocele, testicular atrophy, and genetic disorders such as Klinefelter syndrome, Down syndrome, and McCune-Albright syndrome. The associations with malignancy and infertility remain poorly defined, particularly in the pediatric populations, in which diagnostic challenges and the lack of standardized surveillance protocols complicate management. This review provides an overview of the epidemiology, pathophysiology, and clinical implications of pediatric TM, with a focus on current management practices, surveillance strategies, and areas for future research.

## Introduction

**Figure 1:**
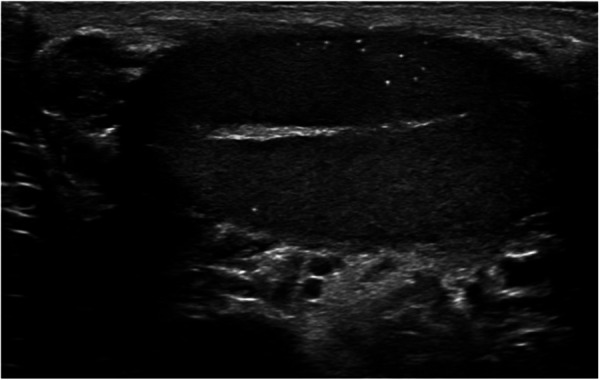
An ultrasound of a patient with TM showing the conditions characteristic punctate calcifications.

Testicular microlithiasis (TM) constitutes small, dispersed calcium deposits within the seminiferous tubules, which may occur in one or both testicles (see Figure 1) (1). These deposits, primarily composed of calcified hydroxyapatite (2), are commonly detected via ultrasonography, where they appear as hyperechoic, diffuse foci that lack acoustic shadowing (3). The microliths have a distinctive, pathognomonic appearance on ultrasound, often described as “innumerable”, “tiny”, or “punctate”, with individual deposits typically ranging from 1 to 3 mm in size (4, 5). TM is usually identified incidentally during imaging for other reasons, as it is generally asymptomatic. Priebe and Garret first described the condition in 1970 when they noted diffuse calcifications on a pelvic x-ray of a 4-year-old boy (6). The first diagnosis of TM using ultrasonography was reported by Doherty et al. in 1987, marking a significant advancement in its detection and sparking increased clinical interest in the condition (7).

Although TM is typically asymptomatic, its discovery has raised concerns about its potential associations with testicular malignancy and infertility. Despite being a relatively rare finding, studies have linked TM to both local benign conditions, such as cryptorchidism, varicocele, and testicular atrophy, as well as systemic genetic disorders like Klinefelter syndrome, Down syndrome, and McCune-Albright syndrome (8). The connection between TM and testicular cancer, however, remains unclear, with conflicting evidence regarding the strength of this association.

The clinical significance of TM in children is an area of ongoing debate. Although it is often considered a benign condition, its potential association with malignancy warrants further investigation to inform best practices and guidelines regarding surveillance. Additionally, the lack of standardized diagnostic criteria and surveillance protocols complicates the management of pediatric TM. Current recommendations for follow-up and surveillance vary, but most suggest patient education and self-examination in patients without risk factors and periodic ultrasound evaluations in those with risk factors (9, 10).

Identification of TM in the pediatric population with significant risk factors may be particularly valuable as established risk factors for testicular cancer tend to increase the likelihood of disease development over time (11). Any further development of screening and monitoring guidelines for pediatric patients with TM, as discussed below, must be carefully weighed with the well-established risks associated with employing any imaging modality, including patient and healthcare-system cost, family emotional stress, and the potential for unnecessary intervention.

Given the conflicting evidence regarding the strength of association between TM and testicular cancer, it is critical to address the unique challenges posed by pediatric TM, including its diagnosis, management, and long-term follow-up. This review aims to provide an overview of pediatric TM, exploring its epidemiology, pathophysiology, and clinical implications. We will also discuss the potential associations with malignancy, examine current approaches to surveillance, and highlight areas where further research is needed to guide clinical practice and improve patient outcomes.

## Diagnosis

TM is painless and impalpable upon physical examination. Thus, TM is typically diagnosed incidentally by scrotal ultrasound. The widespread use of high-resolution ultrasound, the primary diagnostic method for TM, has led to an increased detection rate (12). Scrotal ultrasound of an affected patient typically reveals multiple, non-shadowing echogenic foci that are usually 1 to 3 mm in size, scattered diffusely throughout the testicular parenchyma. This characteristic appearance has gained the moniker “snowstorm” pattern (13).

Diagnosis of TM via ultrasound requires visualization of five or more microliths in a single image, classified as Classical Testicular Microlithiasis (CTM). Conversely, if fewer than five microliths are detected, it is labeled Limited Testicular Microlithiasis (LTM) (14). The echogenic foci seen in TM do not produce acoustic shadowing, distinguishing them from other calcifications that may indicate pathology, such as testicular tumors or infections (3). Although clinical guidelines do not provide differing management strategies for CTM and LTM, patients with CTM are generally at higher risk for associated testicular pathologies than LTM patients (15). Therefore, this classification may be considered among other clinical factors in developing an individualized management plan.

## Prevalence and risk factors

The reported prevalence of TM varies across studies, partly due to differences in the populations examined, the definitions used for TM, and differing ultrasound parameters. For instance, Peterson et al. conducted an extensive study of 1,504 asymptomatic young men with an average age of 22.4 years, reporting a TM prevalence of 5.6% (16). In another large study by Goede et al., involving 694 asymptomatic boys aged 0 to 19, the prevalence was observed at 2.4%—approximately half that found in adults—and noted a trend towards increased prevalence with age. For example, the prevalence of TM was higher in older children and adolescents compared to younger boys (14).

Another potential factor accounting for the discrepancy in these two significant studies was the differences in the radiographic diagnosis for TM. Peterson et al. classified TM as having five or more echogenic foci in a single testis, while Goede et al. defined it as five or more foci in either one or both testes. Although these definitions focus on the count of foci in the testes, other studies in the literature adopt a definition based on the number of foci observed per imaging study (17). Overall, most studies report an incidence between 2.4 and 5.6% in asymptomatic young men.

The prevalence of TM may also differ with the presence of risk factors, as TM is notably higher in patients with specific genetic syndromes like Down syndrome, Klinefelter syndrome (47, XXY), and McCune-Albright syndrome. Vachon et al. reported that 29% of patients with Down syndrome had TM, compared to just 7% in a control group of 200 patients without Down syndrome (*P* < .0001). The increased prevalence of TM in individuals with Down syndrome, along with their higher risk for testicular cancer, suggests a potential link between microlithiasis and malignancy (18).

Similarly, a study by Accardo et al. found that 17.5% of a cohort of 40 Klinefelter patients had TM. However, researchers did not detect a single case of testicular cancer during the follow-up period (19). Other studies have found that boys with Klinefelter syndrome are at an elevated risk for developing germ cell tumors—particularly mediastinal germ cell tumors—compared to the general population (20). However, more extensive studies are needed to determine if this population has an increased absolute risk for testicular malignancy compared to the general population. The relatively low incidence of testicular cancer in Klinefelter syndrome (KS) patients raises questions about the strength of the connection between TM and testicular cancer. However, higher-powered studies are needed to elucidate these associations further.

Another known risk factor for TM is cryptorchidism, as patients with a history of orchidopexy have higher observed rates of TM. Yoshimura et al. found that the incidence of postoperative TM increased over time, reaching 6.0% at 5 years and 11.2% at 10 years after surgery. The study identified a higher testicular position proximal to the external inguinal ring as an independent risk factor for developing TM, with a hazard ratio of 6.18 (21). These findings suggest that patients with a higher testicular position or delayed orchidopexy are at greater risk of developing TM later in life, which parallels the increased risk of malignancy.

Lastly, recent research has found that the prevalence of TM is significantly higher in infertile populations (22, 23), and that TM may adversely affect semen parameters via the obstruction of seminiferous tubules by microliths (24–26). TM is often considered part of a broader condition indicative of underlying testicular dysfunction known as testicular dysgenesis syndrome (TDS), which includes infertility, cryptorchidism, and testicular cancer (22, 27). The association of TM with other conditions that affect testicular development such as hypospadias and varicocele further supports this link (28–30).

## Pathophysiology

The pathophysiology of TM remains somewhat elusive, with many proposed causes, including ectopic oocytes, displaced spermatogonia, and abnormal Sertoli cell activity (1). The pathogenesis may be mediated by environment factors leading to testicular dysgenesis (31), genetic factors (32), and hormonal imbalances that result in poor androgen stimulation of Sertoli cells (33). A study by Nistal et al. investigated eosinophilic bodies and calcifications in children and adults by histochemical and immunochemical methods (34). The study identified non-calcified and partially calcified eosinophilic bodies primarily in children and typically identified completely calcified eosinophilic bodies in adults. Interestingly, the two layers of Sertoli cells typically surrounded the completely calcified microliths within the seminiferous tubules. This finding supports prior theories that degenerated cells unable to be phagocytosed by Sertoli cells within the tubular lumen served as the nidus for microlith formation (35). As part of the proposed mechanism of microlith pathogenesis, glycoprotein then deposits around this cellular debris, and over time the lamellar, concentric rings calcify (1). Researchers theorize that local disturbances in mineral homeostasis may promote the calcification of cellular debris characterizes this mineralization process (36).

Renshaw et al. identified two distinct patterns of calcifications associated with TM (37). The first pattern consists of heterogeneous calcified debris termed “hematoxylin bodies”, which were more specific for germ cell tumors and associated with testicular dysfunction. The second pattern, termed “laminated calcifications” or “corpora amylacea-like bodies”, constitutes organized, concentric layers of calcified material deposited on a central core. This pattern was more prevalent in germ cell tumors, which conferred a higher risk for lymphatic invasion and extension beyond the tunica albuginea.

TM appears to result from a complex interplay of genetic, environmental, and hormonal factors, leading to impaired Sertoli cell function and calcification of intratubular debris. Distinct calcification patterns may reflect differing risks for testicular dysfunction and malignancy, but ultrasound cannot distinguish these patterns.

## TM and association with cancer

Perhaps the most clinically significant aspect of TM is its association with testicular germ cell tumors (TGCTs), a topic of considerable debate. Multiple studies have explored this relationship with varying conclusions.

One of the more established findings regarding TM and TGCTs is the increased prevalence of TM among patients with known TGCTs. A retrospective study by Sharmeen et al. found that 51% of 346 patients diagnosed with primary testicular tumors had at least one microlith, meeting the criteria for limited TM. Additionally, 20% met the criteria for classical TM, with more than five microliths per sonographic image. Interestingly, an increasing microlith count correlated with a lower stage at diagnosis, a higher prevalence of seminoma histology, and a reduced prevalence of embryonal histology (38).

Although the degree to which TM influences the risk for testicular malignancy remains debated, studies have identified specific conditions that strengthen this association. For example, a study by Holm et al. found that among patients with TGCT, those with contralateral TM had a significantly increased risk of carcinoma *in situ* (CIS) in the contralateral testis (odds ratio of 28.6) (39). In a notable pediatric case (40), a patient with TGCT and contralateral TM ultimately developed TGCT in the contralateral testis. A different study by Frandsen et al. found that TM patients with testicular atrophy were significantly more likely to develop germ cell neoplasia *in situ* than TM patients without atrophy (41).

Wang et al. conducted one of the most extensive meta-analyses on TM as a risk factor for testicular malignancy, analyzing 14 studies with a combined total of 35,578 participants (42). Despite significant heterogeneity among the included studies, the study established a strong association between TM and TGCT, reporting a risk ratio of 12.7 (*P* < .001). However, a later meta-analysis by Leblanc et al. presented conflicting results and emphasized the need to control confounding variables by stratifying patients based on risk factors (43). When investigators analyzed asymptomatic patients without risk factors, there was no significant difference in tumor prevalence between those with TM and those without TM. In contrast, among patients with infertility, the tumor prevalence was 22.6% in the TM group compared to 1.7% in the non-TM group, suggesting that TM may be a more meaningful risk factor in select populations. Lack of routine screening in the pediatric population leaves members of these at-risk groups unidentified, posing a challenge to early detection of patients who would most benefit from surveillance.

Trout et al., a study not included in either of these two meta-analyses, constitutes the most extensive multicenter study on the pediatric population (44). The study identified TM in 1,097 of the 37,863 patients, with a prevalence of 2.9%, consistent with prior pediatric estimates. Among boys with TM, 4.64% developed primary testicular tumors, compared to 0.33% of those without TM (unadjusted odds ratio of 14.65). Malignant germ cell tumors were present in 2.8% of boys with TM vs. 0.12% without (unadjusted odds ratio of 22.37). The study concluded that there was a strong association between TM and testicular tumors—particularly TGCT—in the pediatric population.

An even more extensive meta-analysis for the pediatric population was published by Yu et al. just two years later, with a total of 18 studies including 58,195 children. The incidence of TM among children with risk factors for testicular cancer was 2.7%. TM was usually bilateral (69%), of the classic type (71.8%), and with a low rate of new testicular cancer (4/296). Concordantly with prior findings, the study found that TM increased the risk of testicular cancer (risk ratio 15.46). However, researchers did not support routine ultrasound surveillance without additional risk factors (45). These studies emphasize the need for risk stratification and individualized clinical management.

## Management of TM in the pediatric population

Guidelines regarding the management and recommended surveillance of TM remain controversial. Given the typically asymptomatic presentation of TM, in patient's without concomitant risk factors for the development of malignancy the identification of TM may appropriately and broadly classified as a testicular incidentaloma (46). As with any incidentaloma, concern for an inappropriately invasive, expensive, and potentially emotionally tumultuous workup is rightly paramount in recent discussions surrounding appropriate management (47). In contrast, some scholars have also voiced concern that a minimalistic approach to monitoring TM also carries risk, given the relatively understudied relationship between TM and the development malignancy (48).

According to the European Association of Urology (EAU) guidelines, adult patients who have TM and additional risk factors should be informed about their elevated cancer risk and educated on performing regular self-examinations, similar to protocols for patients with a history of undescended testes. The EAU guidelines do not recommend routine sonographic follow-up due to the lack of evidence supporting its efficacy in improving outcomes (45). In the pediatric population, the EAU has based its current guidelines for TM screening practices on a review of 26 studies by Hoen et al., which found that TM in children rarely leads to testicular cancer, with only one case reported among 595 children during follow-up. Since TM may confer a higher cancer risk in adults, especially among those with risk factors, EAU guidelines recommend routine self-examinations from puberty onward for at-risk children (49). Notably, the American Urological Association (AUA) guidelines for clinical monitoring of adults with incidental TM are consistent with the EAU's guidelines for pubertal and post-pubertal patients, recommending against further evaluation of incidentally identified TM unless other risk factors for malignancy are concurrent (i.e., cryptorchidism, personal or family history of testicular malignancy) (48).

The European Society of Urogenital Radiology (ESUR) subcommittee on scrotal imaging published guidelines on the imaging and surveillance of testicular microlithiasis and includes recommendations for the pediatric population. Key risk factors that influence management include a past medical history of TGCT, undescended testes (maldescent), prior orchiopexy, testicular atrophy, and a family history of TGCT in first-degree relatives, which are all independent risk factors for testicular malignancy. These guidelines recommend a more nuanced approach for the adult population and provide specific criteria to guide management, including the density of microliths. For example, in cases of diffuse TM or extensive microliths obscuring the testicular echotexture, annual scrotal ultrasound is advised even without risk factors. For the pediatric population, the guidelines do not specify if the density of microliths on imaging should influence management like the adult population. However, the presence of the listed risk factors warrants annual ultrasound surveillance and parental education regarding scrotal examinations. Patients with associated syndromes such as Klinefelter's syndrome and McCune-Albright syndrome should be treated similarly to the general population due to the lack of evidence that these patients have a significantly higher risk for TGCT compared with patients with TM alone (10).

The guidelines do not address surveillance with serum tumor markers (STMs) in these patients, and current research on this screening modality does not demonstrate any benefit in the asymptomatic pediatric patient with TM (50).

The ESUR guidelines also specifically mention fast-track ultrasound (US) access as a crucial component in managing patients with TM who are discharged with guidance on self-examinations. Fast-track US access ensures that clinicians can promptly evaluate any new, concerning findings identified during self-examinations can be promptly evaluated. Fast-track pathways minimize diagnostic delays and alleviate the burden on primary care by allowing direct access to US imaging without the need for repeated referrals (8).

Our institution tends to favor the ESUR guidelines. While these guidelines do not recommend routine US for isolated TM, they support annual US follow-up in high-risk patients. This approach may be beneficial in identifying early changes that might not be palpable during self-examination. Moreover, maintaining regular follow-up can enhance patient engagement and ensure adherence to self-examinations.

The psychological impact of a TM diagnosis can be profound for both the patient and their family, as well as to providers. In a recent study of the management of small (<1 cm), non-palpable testicular incidentalomas published by Bertolotto et al.,11 out of 77 of non-growing lesions identified were operated upon per patient or provider preference; 10 of these lesions were non-neoplastic, and one was a benign tumor (51). These results replicate similar findings in a previous study, Toren et al. (52). While not perfectly applicable to the pediatric population with TM, these startling results do imply the potential cost that over-diagnosis and over-screening in TM may confer to patients and their families. Similar studies directed at interventions and outcomes specific to the pediatric patient population with TM are needed, and would provide more concrete evidence to inform providers, patients, and families in their approach to TM.

Despite its generally benign nature, the association between TM and testicular cancer can lead to heightened anxiety and have the potential to lead to unnecessary intervention. Concerns about fertility implications and the burden of ongoing monitoring and self-examinations can create emotional stress as well. Providing clear education, supportive counseling, and reassurance about the low malignancy risk in childhood is important in reducing these effects (53).

## Future directions

Despite the growing body of research on TM, several key questions remain unanswered. Although the data demonstrate an increased risk for testicular malignancy in the post-pubertal TM population, there is still conflicting evidence of any significant risk in the pre-pubertal population. Understanding this question can help determine the utility of surveillance of pre-pubertal children in select populations. Additionally, the role of genetic factors in the development of TM and how they might influence the progression to testicular cancer remains an area of interest for future basic science research.

Despite the EAU and ESUR guidelines discussed above, there remains a lack of consensus on the need for routine surveillance with ultrasound or serum tumor markers for children with TM and risk factors, and the indications for follow-up remain unclear. Long-term studies assessing the natural course of TM in pediatric patients and the outcomes of various management strategies are needed to provide more explicit guidance on management. The potential for using biomarkers or genetic testing to predict better the risk of malignancy in high-risk TM patients might be a fruitful avenue for future research.

In conclusion, TM requires careful consideration in pediatric populations due to its associations with testicular malignancy. While often benign, TM is frequently present in individuals with risk factors such as personal or family history of testicular malignancy, cryptorchidism, and testicular atrophy, which warrant more vigilant surveillance. Although recommendations for management differ, clear patient education, guidance on self- or parental examination, and fast-track US access are important for detecting malignancy early for any patient with risk factors. Ultimately, more research in the pediatric population is needed to develop standardized, evidence-based guidelines to optimize outcomes and minimize unnecessary interventions.
